# Monolithic integration of nanorod arrays on microfluidic chips for fast and sensitive one-step immunoassays

**DOI:** 10.1038/s41378-021-00291-w

**Published:** 2021-08-17

**Authors:** Ye Wang, Jiongdong Zhao, Yu Zhu, Shurong Dong, Yang Liu, Yijun Sun, Liling Qian, Wenting Yang, Zhen Cao

**Affiliations:** 1 College of Information Science and Electronic Engineering, Zhejiang University, 310027 Hangzhou, People’s Republic of China; 2 Suzhou Institute of Nano-tech and Nano-Bionics, Chinese Academy of Sciences, 215123 Suzhou, People’s Republic of China; 3 Hangzhou Global Scientific and Technological Innovation Center, Zhejiang University, 310018 Hangzhou, People’s Republic of China; 4 Children’s Hospital of Fudan University, Shanghai, People’s Republic of China; 5 Genenexus Technology Corporation, Shanghai, People’s Republic of China

**Keywords:** Nanorods, Plasmonics, Immunoassay, Oblique angle deposition, Biosensors, Biosensors

## Abstract

Here, we present integrated nanorod arrays on microfluidic chips for fast and sensitive flow-through immunoassays of physiologically relevant macromolecules. Dense arrays of Au nanorods are easily fabricated through one-step oblique angle deposition, which eliminates the requirement of advanced lithography methods. We report the utility of this plasmonic structure to improve the detection limit of the cardiac troponin I (cTnI) assay by over 6 × 10^5^-fold, reaching down to 33.9 fg mL^−1^ (~1.4 fM), compared with an identical assay on glass substrates. Through monolithic integration with microfluidic elements, the device enables a flow-through assay for quantitative detection of cTnI in the serum with a detection sensitivity of 6.9 pg mL^−1^ (~0.3 pM) in <6 min, which was 4000 times lower than conventional glass devices. This ultrasensitive detection arises from the large surface area for antibody conjugation and metal-enhanced fluorescent signals through plasmonic nanostructures. Moreover, due to the parallel arrangement of flow paths, simultaneous detection of multiple cancer biomarkers, including prostate-specific antigen and carcinoembryonic antigen, has been fulfilled with increased signal-to-background ratios. Given the high performance of this assay, together with its simple fabrication process that is compatible with standard mass manufacturing techniques, we expect that the prepared integrated nanorod device can bring on-site point-of-care diagnosis closer to reality.

## Introduction

Immunoassays have been widely explored and have become the gold standard for detecting myriad biomarkers in disease treatment and clinical diagnostics^1,2^. It employs specific bindings of antibodies and antigens for quantitative analysis of proteins in biological samples. Fluorescent immunoassays are one of the most popular and well-developed analytical methods that measure fluorescence to quantify target antigens in clinical laboratories^3,4^. However, conventional fluorescent immunoassays are routinely performed in 96-well plates, require complicated operating procedures, have long incubation times, and need bulky instruments, which are incompatible with the requirements of on-site diagnostics and point-of-care testing^5^. In recent years, the advances in lab-on-a-chip technology have contributed to the progress of miniaturized bioanalytical devices^6–8^. Many portable, rapid, and cost-effective microfluidic chips have been developed to address special issues in fluorescent assays, such as labor-intensive preparation procedures, nonspecific binding, or difficulty in miniaturization. For instance, Gervais and Delamarche proposed a plastic microfluidic chip for a one-step immunoassay^9^. By integrating various fluidic components and reagents on chips, inflammation and the cardiac marker C-reactive protein (CRP) were detected at a concentration <1 ng mL^−1^ in 5 min. Song et al. presented a multiplexed volumetric bar-chart chip for the rapid quantification of protein biomarkers with a simple operation and easy read-out^10^. They further utilized gas competition and reported a digital volumetric bar-chart chip (DV-chip) for quantitative diagnostics with ultralow detection limits (~0.1 pM) that are readable with the naked eye^11^. Other microfluidic concepts for miniaturized bioassays include lab-on-paper^12–14^, lab-on-a-disc^15–17^, lab-on-a-syringe^18^, and lab-on-a stick^19^.

Despite substantial progress made toward the improvement of assay throughput, decreases in costs and time, and operational simplification, the sensitivity or detection limit remains one of the core parameters that can be tremendously increased using nanostructures or nanoparticles due to the metal-enhanced fluorescence (MEF) effect. With the rapid development of nanotechnology, various nanostructures are fabricated and dramatically improve the sensitivity of immunoassays. Typical examples include plasmonic gold-on-gold (Au/Au) films^20^, nanoparticles^21,22^, nanopillars^23–25^, nanorods^26,27^, and nanowells^28^. Among those techniques, advanced nanofabrication techniques such as e-beam lithography (EBL) or nanoimprinting are commonly employed to pattern the desired nanostructures on the substrate. However, EBL is limited by expensive equipment and suffers from low throughput. Nanoimprinting may involve complicated preparation of the imprint template and raise issues such as mold deformation during the process^29^. Moreover, colloidal lithography is proposed using the self-assembly of colloidal particles as etching masks for nanopatterning^30^. Nevertheless, it requires a cumbersome etching process and may face issues such as its uniformity over large areas. Bottom-up solution synthesis is not suitable for mass production and suffers from complex operating procedures. Therefore, it is of utmost importance to find effective nanostructures with easier fabrication schemes to improve the MEF and detection limit of assays.

Previously, we reported the easy fabrication of nanorod arrays through oblique angle deposition (OAD), and these nanostructures have been extensively studied and applied in many fields, such as surface-enhanced Raman spectroscopy^31,32^ and high-performance capillary electrophoresis^33,34^. Herein, we present a simple and sensitive microfluidic immunoassay chip that integrates Au nanorods in microchannels for the quantitative detection of cardiac troponin I (cTnI). These nanostructures are selectively immobilized on the detection zone of the chip through one-step evaporation, which eliminates the requirement for advanced lithography tools or complicated processes. The plasmonic substrates afford fluorescence enhancement and provide a large surface area for antibody conjugation. The sensitivity of the cTnI assay is greatly improved by over 6 × 10^5^-fold, with a detection limit down to 33.9 fg mL^−1^ (~1.4 fM), compared with an identical assay on glass plates. Moreover, the fluidic chips monolithically integrated with Au nanorods enable a flow-through immunoassay for cTnI within 6 min with a decrease in the detection limit from 27.0 ng mL^−1^ (~1.1 nM) down to 6.9 pg mL^−1^ (~0.29 pM). A layout of multiplex flow paths is also designed for the simultaneous detection of highly sensitive cancer biomarkers prostate-specific antigen (PSA) and carcinoembryonic antigen (CEA).

**Fig. 1:**
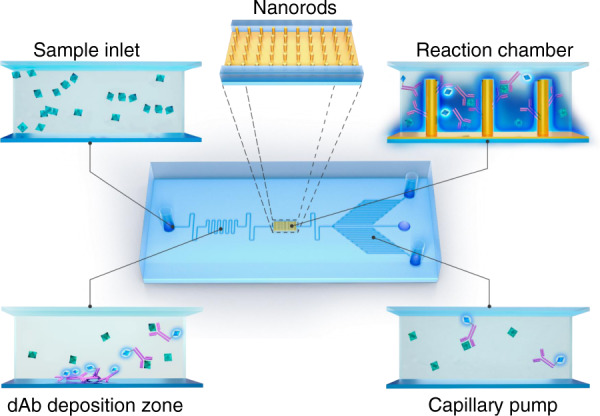
Conceptual illustration of the flow-through microfluidic device integrated with Au nanorods for one-step immunoassay of protein biomarkers. The serum sample containing target biomarkers is introduced and conjugated with the fluorescent dAb prepatterned in the deposition zone. The complex flows through the reaction chamber and is captured by cAb conjugated with dense arrays of embedded Au nanorods. The enhanced fluorescent signals are measured and analyzed through a microscope equipped with a CCD camera. The flow rate and total reaction time are defined by the capillary pump

The conceptual design of our fluidic chip integrated with nanorods is shown in Fig. 1 for highly sensitive immunoassays. The sample is introduced from the inlet and pumped through the flow resistor to the detection antibody (dAb) zone, where the fluorescent dAb is predeposited on the glass substrate. The target antigen in the sample binds with the dAb as it flows to the reaction chamber. In this chamber, the complex is bound to the capture antibody (cAb), which is covalently conjugated with dense arrays of embedded nanorods. The fluorescent signal is boosted due to MEF and detected by microscopy for further analysis.

## Results and discussion

### Simulations

**Fig. 2:**
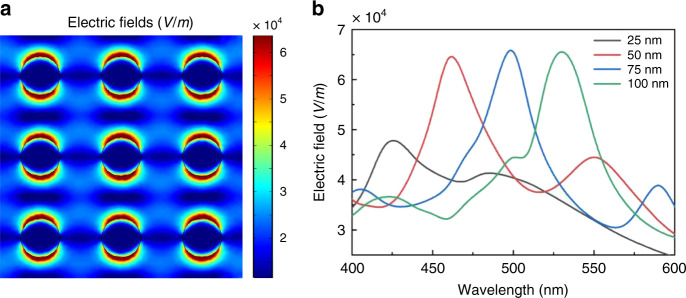
Simulation of the enhanced electric fields around Au nanorods. **a** Numerical simulation plots showing the electric field distribution over the translational cross section of the Au nanorod arrays with 75 nm diameters and 200 nm pitches. In the configuration, the wavelength of the plane light source is set to 493 nm, similar to the fluorescence emission wavelength of Alexa 488. **b** Spectrum of the maximum electric fields for the Au nanorod arrays with various diameters

**Fig. 3:**
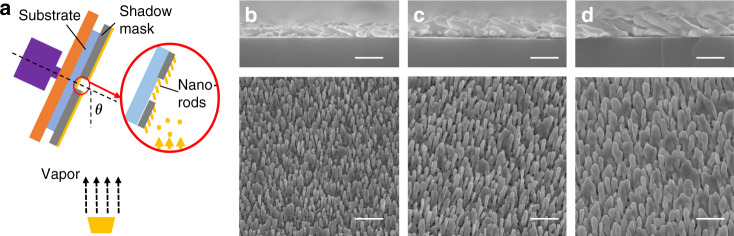
Nanorod fabrication through oblique angle deposition (OAD). **a** Rendering of the principle using OAD to integrate nanorods on the specific region of glass substrates with a shadow mask. **b**–**d** Scanning electron micrographs of the cross-sectional and top views of the Au nanorods with nominal thicknesses of **b** 1 μm, **c** 1.5 μm, and **d** 2 μm (all scale bars: 500 nm)

MEF refers to the phenomenon that fluorescence emissions of fluorophores are significantly increased when they are in close proximity to conducting metallic particles, colloids, or surfaces. This is mainly due to the strong and close-range interactions between these fluorescent molecules with intensified local electric fields around the nanostructures by localized surface plasmon resonance (LSPR), which enhances fluorescence absorption, raises the quantum yield, and increases the radiative decay rate^35^. To explore the MEF performance of nanorods, theoretical simulations were carried out using COMSOL Multiphysics. Figure 2a shows the field distribution of ~75-nm-diameter Au nanorod arrays with 200 nm pitches to mimic the detailed structures illustrated in Fig. 3c. We chose Au nanorods rather than Ag for their better stability and subsequent conjugation with biomolecules. The polarized beam is incident onto the nanorods from the top. High enhancement of the electric fields can be observed at the adjacent regions of the nanorods due to the strong LSPR and then exponentially decay. This suggests that the coupling between the excited states of the fluorophores and surface plasmons of nanorods occurs mainly when they are located within ~10 nm of each other. The wavelength dependence of the maximum electric field intensity for nanorods with various diameters is shown in Fig. 2b. A redshift in the spectrum was observed with an increase in the nanorod diameter (from 25 to 100 nm). Simulation of the spectrum of maximum electric fields for Au nanorod arrays with a 75 nm diameter but various pitches was also conducted (Supplementary Fig. S1a). The results show that the resonance peak redshifts and slightly decreases with an increase in the pitches (from 200 to 400 nm). According to the simulation results, the geometry of the nanorods was optimized to 75 nm in diameter and 200 nm pitches, making its plasmonic resonance close to the excitation and emission peaks of fluorescent dye (Alexa 488, ex/em 493/519 nm) normally used in immunoassay experiments. To confirm this, the absorbance of the fabricated nanorod arrays with a 75 nm diameter was obtained by measuring the transmission and reflection spectrum of the sample (Supplementary Fig. S1b). The resonance peak of absorbance was measured to be 0.98 at 495 nm with a full-width at half-maximum of 120 nm for the optimized nanorod arrays.

### Morphology

Dense arrays of nanorods were integrated into the designed region of glass substrates using OAD with a shadow mask (Fig. 3a), eliminating the requirement for expensive advanced lithography. Briefly, it involves large incident angle deposition of metal atoms with respect to the surface normal of the substrate. The initial nucleation of atoms favors the further growth of nanorods due to the shadowing effect. Figure 3b–d reveals the morphologies of the prepared Au nanorods with distinct nominal thicknesses on the substrates. On the substrates that received 1-μm-thick Au evaporation, the discrete nanorods grew independently in the direction of deposition at a tilting angle of ~15° with respect to the surface normal. This tilting angle of nanorods can be eliminated if a rotation of the substrate is applied during evaporation. It should be mentioned that the tilting angle has little influence on the subsequent fluorescent enhancement and assay performance. The average diameter and length according to the scanning electron microscopy (SEM) images were measured to be 59.9±9.2 nm (*n* = 10) and 376.0 ± 52.4 nm (*n* = 10), respectively. As the nominal deposition thickness increases, the nanorods tend to fuse together and are difficult to resolve individually. The nanorods resulting from a nominal thickness of 1.5 μm exhibit an average diameter of 77.1 ± 8.0 nm (*n* = 10) and a length of 608.8 ± 10.2 nm (*n* = 10), while the nanorods that received a deposition of 2 μm nominal thickness are 103.6 ± 19.6 nm (*n* = 10) in diameter and 682.7 ± 38.1 nm (*n* = 10) in length. Notably, compared with those patterned through standard microfabrication techniques such as EBL or nanoimprinting, the nanorods fabricated by OAD demonstrate more random irregularities, such as protrusions, bifurcations, and fusion, as suggested in the SEM images (Fig. 3b–d). These irregularities of the Au nanorod surfaces can generate more hot spots for electric field enhancement, as previously revealed by both theoretical calculations and experiments^36,37^.

### Sub-femtomolar sensitivity

**Fig. 4:**
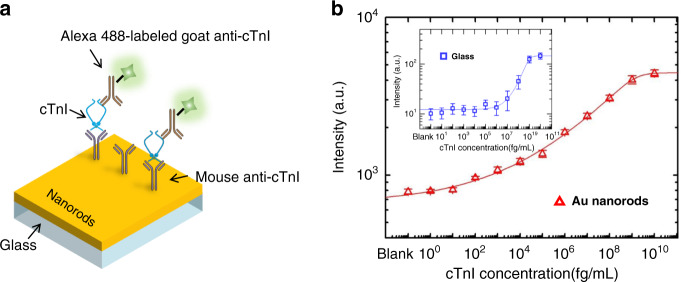
The cTnI assay with sub-femtomolar sensitivity on Au nanorod substrates. **a** Conceptual illustration of the cTnI assay performed on Au nanorod arrays. **b** Dilution plot of fluorescent intensity against the concentration of target cTnI on both Au nanorod arrays (red triangles) and reference glass plates (inset, blue squares). The curves were fitted to a five-parameter logistic nonlinear regression model with an excellent agreement (*R*^2^ = 0.998). The error bars indicate the standard errors of the respective mean values based on five measurements

The detection sensitivity of conventional immunoassays can be significantly improved due to the enhanced fluorescent signals and more surface sites for antibody binding. To test assay performance, we performed immunoassays of the clinically relevant protein biomarker human cTnI, one of the most crucial markers for acute myocardial infarction, using nanorod substrates. A schematic description of the assay protocols is depicted in Fig. 4a. The nanorod arrays were first functionalized with a self-assembled monolayer (SAM) of 3,3′-dithiodipropionic acid di(*N*-hydroxysuccinimide ester) (DSP) and then covalently immobilized with monoclonal mouse anti-cTnI. Following the washing and blocking step, the target cTnI was serially diluted to various concentrations and incubated with the substrates for 2 h. For detection, goat polyclonal anti-cTnI conjugated with Alexa 488 was used to produce sandwich complexes and for fluorescent identification. The SAM of DSP was ~1.5 nm thick with a measured refractive index of 1.40. With a cAb layer of ~4.10 nm and a target antigen layer of ~3.76 nm^38^, the total spacer thickness was estimated to be ~9.36 nm, which was in the range of strong LSPR. The resulting logarithm plot of fluorescent intensity for target cTnI ranging from 1 fg mL^−1^ to 10 μg mL^−1^ is shown in Fig. 4b. The curves were fitted to a five-parameter logistic nonlinear regression model and showed excellent agreement with an *R*^2^ value >0.99. The limit of detection (LoD) value was derived from the concentration that generates a signal equal to three times the standard deviation (SD) from the mean value of the blank sample measurement, given that all these measurements follow a Gaussian distribution. The sandwich assay of cTnI on substrates integrated with gold nanorod arrays demonstrated a detection sensitivity down to 33.9 fg mL^−1^ (~1.4 fM), together with a dynamic range of over six orders of magnitude (from 2.3 pg mL^−1^ to 1 μg mL^−1^). For comparison, identical cTnI sandwich immunoassays were also conducted on glass slides modified with poly-l-lysine (PLL). The LoD of these immunoassays was found to be 22.9 ng mL^−1^ (~1.0 nM), and the dynamic range was ~3 orders of magnitude. Therefore, the plasmonic resonance and the associated enhanced fluorescent signals afforded by the dense nanorod arrays improved the sensitivity by over 6 × 10^5^-fold and broadened the dynamic range relative to the reference glass plates. It is plausible that assay performance using other fluorescent dyes with distinguishing excitation and emission wavelengths could also be boosted through engineering nanorod morphology via OAD. In addition to cTnI, other biomarkers, including CEA and PSA, were also detected using similar assays on both nanorod array substrates and the reference glass slides (Supplementary Fig. S2). The achieved specifications of assay performance are summarized in Table 1. The LoD of the PSA assay on Au nanorods attained 36.6 fg mL^−1^ (~1.2 fM), referring to 29.0 ng mL^−1^ (~1.0 nM) on glass substrates, while the CEA assay on Au nanorods yielded a 5 × 10^5^-fold sensitivity increase with a detection limit from 98.9 ng mL^−1^ (~1.4 nM) down to 194.3 fg mL^−1^ (~2.7 fM). The sensitivity of our device is comparable to that of state-of-the-art devices fabricated through standard methods. For example, Tabakman et al. presented nanostructured gold films prepared by complicated solution methods for enhancement of the detection limit down to ~5 fM^20^. Zhou et al. reported the use of nanorod arrays and achieved a detection sensitivity of immunoglobulin G (IgG) down to 0.3 fM^25^. The plasmonic structures were patterned through nanoimprinting and reactive ion etching, which required preparation of the imprint mold and cumbersome etching processes.

**Table 1:** Specification (limit of detection, dynamic range, and CV) of cTnI, PSA, and CEA assays on Au nanorod arrays and glass plates

Biomarkers	Samples	Limit of detection	Dynamic range	CV%^a^
cTnI	Blank glass	22.9 ng mL^−1^	50.7 ng mL^−1^–10 μg mL^−1^	15.2
	Au nanorods	33.9 fg mL^−1^	2.3 pg mL^−1^–1 μg mL^−1^	11.4
PSA	Blank glass	29.0 ng mL^−1^	73.6 ng mL^−1^–10 μg mL^−1^	16.7
	Au nanorods	36.6 fg mL^−1^	1.6 pg mL^−1^–1 μg mL^−1^	13.2
CEA	Blank glass	98.9 ng mL^−1^	264.7 ng mL^−1^–10 μg mL^−1^	19.1
	Au nanorods	194.3 fg mL^−1^	6.2 pg mL^−1^–1 μg mL^−1^	10.8

^a^CV refers to the interassay coefficients of variability on five different samples.

### Flow-through assay

**Fig. 5:**
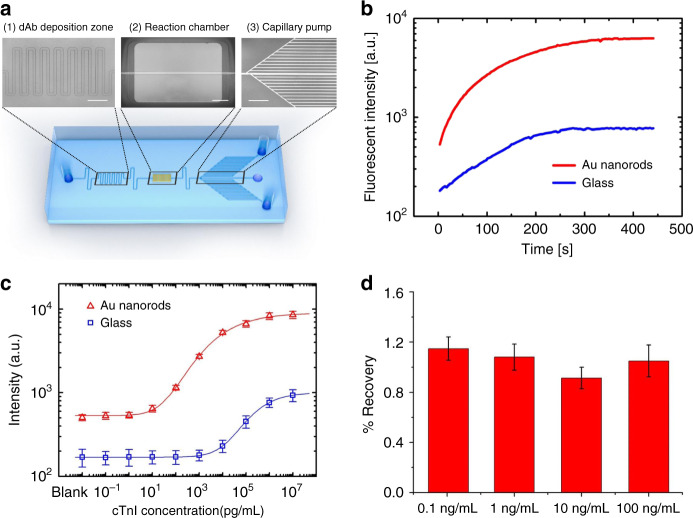
One-step flow-through immunoassay of cTnI with integrated Au nanorods. **a** Picture of a representative flow-through immunoassay chip containing (1) a dAb deposition zone, (2) a reaction chamber with integrated Au nanorods, and (3) a capillary pump (all scale bars: 500 μm). **b** Time evolution of fluorescence intensity in the reaction chamber on both Au nanorods and glass substrates. **c** Calibration curves for cTnI quantification generated by averaging the fluorescence intensity of the reaction chamber region using a flow-through assay device with and without Au nanorods. **d** Recovery rate for the typical concentrations of cTnI spiked in human serum. All error bars are based on five repetitious measurements

For on-site point-of-care diagnostics, it is of utmost priority that a fast and highly sensitive assay platform is developed with a simple handling procedure, low sample assumption, rapid reaction time, and accurate and sensitive detection results. Herein, we adopted a flow-through microfluidic assay device monolithically integrated with our dense nanorod arrays for quantitative immunoassays (Fig. 5a). In our design, the dAb conjugated with Alexa 488 was prepatterned in the deposition zone by pipetting 20 μL of solution in the stencils and incubating for 2 h at room temperature. The cAb mouse anti-cTnI was preimmobilized on the Au nanorods through a monolayer coating of DSP (see the “Materials and methods” section for details). Human serum spiked with various concentrations of target antigens was introduced through the sample inlet. When it reached the dAb deposition zone, the dAb labeled with fluorescence dissolved into the stream of the sample flow and bound with the target to form the antibody–antigen complex. A serpentine microchannel design was employed for chaotic advection that rapidly mixes the dAb and target. The complex was transported toward the reaction chamber and captured by the cAb on the nanorods. The fluorescent signal could be enhanced through MEF of these plasmonic nanorod arrays to assure a low detection limit. The unreacted residues were washed away from the reaction chamber to the waste reservoirs. The capillary pump consists of small parallel microchannels, which determine the main capillary pressure on the liquid and therefore define the average flow rate. The measured average flow rate here was 0.6 nL s^−1^, while the total filling time required was ~6 min. The filling velocity and maximum volume of the sample consumed in the assay could be adjusted through modification of the number and dimension of the microchannels in the capillary pump. It should be noted that all washing steps occasionally used in conventional 96-well immunoassays were eliminated here by a flow-through process.

To demonstrate the clinical utility of our flow-through assay devices integrated with nanorods, we performed an assay to detect cTnI spiked in human serum. The measurement of fluorescence intensity in the reaction chamber over time revealed that the signal continually increased as the dAb–antigen complex passed through the reaction chamber and reached a plateau after 5 min (Fig. 5b). A series of samples with different concentrations were tested using separate devices, and the calibration curve is shown in Fig. 5c, suggesting a proportional relationship between the fluorescent strength and logarithmic concentrations ranging from 1 to 1000 ng mL^−1^. The LoD reached as low as 6.9 pg mL^−1^ (~0.3 pM), and the dynamic range reached nearly 3 orders of magnitude (from 34.3 pg mL^−1^ to 10 ng mL^−1^). For reference, the LoD of the flow-through assay performed on devices with no nanorods was 27.0 ng mL^−1^ (~1.1 nM). In this case, fluorescent enhancement by nanorods afforded a 4000-fold increase in the detection limit compared with bare glass substrates. Compared with the femtomolar sensitivity achieved by the assays in the device with the same nanorods, but not in a flow-through manner, the LoD reported here was found to be 2 orders of magnitude higher. This could be mainly attributed to the insufficient incubation time and omission of multiple washing processes, which might lead to unspecific binding and large background noise. Nonetheless, this one-step immunoassay device notably simplifies the procedure of conventional diagnostic methods, and the total detection time is <6 min, while the assay sensitivity is remarkably improved by 4000-fold compared with bare glass devices. The performance is on par with those from state-of-the-art flow-through assay devices at comparable test times and yet achieved a relatively higher sensitivity. For instance, Gervais and Delamarche demonstrated capillary-driven microfluidics for effective one-step immunoassays with a detection limit of 1 ng mL^−1^ CRP in 14 min^9^. Yu et al. fabricated a dextran-modified polydimethylsiloxan (PDMS) microfluidic enzyme-linked immunosorbent assay (ELISA) device and simultaneously detected multiple biomarkers, including IL-5, HBsAg, and IgG^39^. The device showed an LoD of 100 pg mL^−1^ and a dynamic range of 5 orders of magnitude. Various nanostructures were also incorporated with microfluidic devices for fast and sensitive assays. Hu et al. utilized glass capillaries decorated with zinc oxide (ZnO) nanorods for multiple detections of PSA, alpha-fetoprotein, and CEA in the serum^40^. The detection limits were reported to be 1–5 ng mL^−1^ and achieved within 30 min. Sang et al. demonstrated ZnO nanowires grown in microfluidic channels for an anti-mouse IgG assay with a detection limit of 4.17 pM^41^. However, these nanorods were prepared through a complicated solution method that was incompatible with mass manufacturing processes. Li et al. reported a DV-chip that utilizes platinum nanoparticles as the ELISA probe for easy and fast on-site assays. The minimum differences of CEA samples between 1 and 1.5 ng mL^−1^ could be recognized by the device^11^. Alternatively, lateral flow immunoassays (LFAs) have also presented considerable advantages and have been widely applied as scaffolds for protein immunoassays. Although it proved to complete the reaction in a relatively short time and eliminated the requirement for sophisticated read-out systems, standard LFAs usually suffer from poor detection limits (~10 pM or 100 pg mL^−1^)^42,43^. Some signal-enhancing materials and new labeling methods have been employed to tackle this challenge, including quantum dots^44,45^, carbon nanoparticles/nanotubes^46^, and magnetic nanoparticles^47^. However, the complicated sample preparation processes and additional liquid handling steps hindered their practical application. It is highlighted that our integrated nanorod devices, with their extraordinary assay performance, are easily fabricated through one-step OAD without resorting to expensive nanofabrication equipment.

The accuracy of our assay platform was also examined through recovery tests. Representative concentrations (0.1, 1, 10, and 100 ng mL^−1^) of cTnI were added to blank human serum and detected using flow-through assay devices integrated with Au nanorods. The expected concentrations were determined on the basis of calibration curves and measured fluorescent intensities. As shown in Fig. 5d, the percent recovery rates for the four samples were 114, 108, 91, and 105%, all within the acceptable range from 80 to 120%. Based on the results, this assay platform is sufficiently reliable for the quantification of cTnI in biological samples.

### Parallel assay

**Fig. 6:**
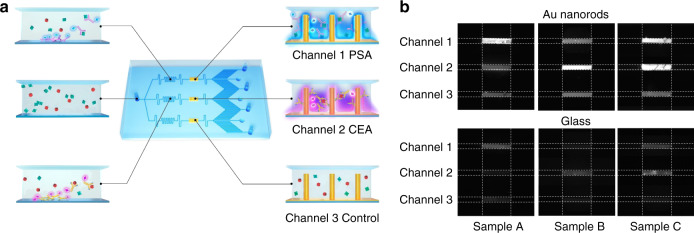
Parallel assay of PSA and CEA using flow-through devices integrated with Au nanorods. **a** Schematic description of the flow-through immunoassay device integrated with Au nanorods for parallel biomarker quantification. The sample fluid was split into three streams passing over reaction chambers immobilized with distinct cAbs for detection of the cancer biomarkers PSA and CEA. **b** Fluorescent images of the reaction chambers on flow-through devices with Au nanorods (upper panel) and glass substrates (lower panel) after detecting serum samples containing solely 1 ng mL^−1^ PSA (sample A), 1 ng mL^−1^ CEA (sample B), or the mixed PSA/CEA with an equal concentration of 1 ng mL^−1^ (sample C)

Furthermore, by the parallel arrangement of the sample flow paths and immobilization of different antibodies, multiple biomarkers can be simultaneously detected at high sensitivity. As shown in Fig. 6a, the fluid of the sample containing various target proteins was guided into a plurality of microchannels 100 μm wide and 45 μm high and captured by manifold-functionalized nanorods in disparate reaction chambers. As a proof-of-concept, a three-channel device was fabricated and precoated with corresponding probes for the parallel detection of cancer biomarkers of PSA (Channel 1) and CEA (Channel 2). Channel 3 served as a negative control whereby no cAb was conjugated with nanorod arrays. The pressure of the capillary pump was characterized as −32 Pa, and the flow rate was measured as 2 nL s^−1^. The required filling time was ~5 min. Serum samples containing solely 1 ng mL^−1^ PSA (sample A), 1 ng mL^−1^ CEA (sample B), or the mixed PSA/CEA with an equal concentration of 1 ng mL^−1^ (sample C) were loaded into individual devices with nanorods and analyzed for the fluorescence intensity of the assays (Fig. 6b, upper panel). The results revealed that no obvious fluorescent change was observed in the negative control of Channel 3, indicating marginal nonspecific binding. For sample A or B, only Channel 1 or 2 displayed the expected positive fluorescence intensity, while Channels 1/2 demonstrated positive signals for sample C, indicating good agreement in antigen–antibody reactivity. For reference, planar glass devices without nanorods were also fabricated and tested, as shown in Fig. 6b (lower panel). Despite the expected positive and negative responses, the signal-to-background ratios obtained were significantly lower than those of the integrated nanorod devices. The calibration curves over the concentration suggested a detection limit of 2.9 pg mL^−1^ (~0.1 pM) for PSA and 131.4 pg mL^−1^ (~1.8 pM) for CEA on Au nanorod devices, compared with 3.7 ng mL^−1^ (~0.1 nM) and 55.8 ng mL^−1^ (~0.8 nM) on glass devices for PSA and CEA, respectively (Supplementary Fig. S3). Unambiguously, these results confirmed that the fluorescence enhancement afforded by the Au nanorod arrays increased the sensitivity and broadened the dynamic range for immunoassays. We further carried out experiments that introduced the sample containing only CEA into the flow-through devices with nanorods conjugated with anti-PSA to test the potential cross-reactivity of the anti-PSA antibodies toward CEA (Supplementary Fig. S4a). As shown in Fig. S4a, the acquired fluorescent signals obtained by introducing 1 ng mL^−1^ CEA sample into the flow-through nanorod devices conjugated with anti-PSA were not significantly different from those of the blank samples, indicating no cross-reactivity of the anti-PSA toward CEA. Similarly, it was confirmed that there was no cross-reactivity between the anti-CEA and PSA (Supplementary Fig. S4b). Moreover, the obtained signals for a 1 ng mL^−1^ PSA sample were visibly stronger than those of the negative control (Fig. 6b and Supplementary Fig. S4a), which implied that the binding of nonspecific proteins was suppressed to avoid false-positive results. It is worth mentioning that the nanorods integrated into parallel reaction chambers were fabricated with the same parameters optimized for the same fluorescent labels of dAb. For real assays involving different fluorescent labels, further optimization of nanorod structure parameters is required, as well as shadow masks to fabricate various nanorods on selected regions of the substrates. Nevertheless, with its easy fabrication process and high assay performance, our microfluidic immunoassay devices integrated with dense arrays of nanorods have a high potential for application in on-site high-throughput screening for many diseases.

## Conclusion

In summary, we demonstrated a facile and easy method to monolithically integrate Au nanorods in microfluidic devices for flow-through immunoassay detection of biomarkers. These dense arrays of nanorods demonstrated fluorescence enhancement effects and therefore result in high-performance assays with considerable improvement in their sensitivity and dynamic range. Through scientific simulation, we shed light on the underlying mechanism for MEF and accordingly optimized shape profiles for the designed sandwich assay. Leveraging on this device, we achieved a one-step flow-through assay for the cardiac protein biomarker cTnI with a detection limit down to 6.9 pg mL^−1^ (~0.3 pM) in <6 min, 4000 times lower than conventional glass substrates. Furthermore, by the parallel arrangement of flow paths, the parallel detection of the cancer biomarkers PSA and CEA was accomplished with enhanced signal-to-background ratios. The monolithic integration of nanorods does not require advanced lithography tools and can be further scaled up for application in the on-site point-of-care diagnosis of myriad diseases. Investigations are currently underway for portable smartphone-based fluorimeters for real point-of-care diagnostic applications.

## Materials and methods

### Nanorods by OAD

OAD was performed to integrate nanorods on the wafer. In brief, the substrates were placed on the stage of an e-beam evaporator and adjusted to a large tilt angle *α* = 85° with respect to the surface normal. A 5-nm-thin film of titanium was first deposited at a rate of 2 Å s^−1^ as an adhesive layer. Subsequently, a layer of Au nanorods was deposited at *α* = 85° and **r** = 3 Å s^−1^. The stage was under vacuum (<10^−7^ torr) for all the depositions.

### Reagents

Capture antibodies, including monoclonal mouse anti-cTnI (clone# 19C7; HyTest Ltd, Shanghai), monoclonal mouse anti-PSA (clone# 8A6; HyTest Ltd, Shanghai), and monoclonal mouse anti-CEA (clone# 3C1; HyTest Ltd, Shanghai), were diluted to a final concentration of 10 μg mL^−1^ in 1× phosphate-buffered saline (PBS) coated on the Au nanorods through a SAM of DSP (Sigma-Aldrich, USA). Bovine serum albumin (BSA, 1% (w/v), Sigma-Aldrich, USA) was used as a blocking buffer to reduce nonspecific binding. For reference glass chips, 3% (w/v) PLL (Sigma-Aldrich, USA) in PBS was utilized as the crosslink between proteins and silicon oxide. The target antigens of cTnI, PSA, and CEA purchased from Abcam (Cambridge, UK) were spiked with human serum (cTnI, PSA, and CEA free) to different concentrations and loaded into the reservoirs for testing. Fluorescent Alexa 488-labeled goat polyclonal anti-cTnI, goat polyclonal anti-PSA, and goat polyclonal anti-CEA purchased from Abcam (Cambridge, UK) were employed as detection antibodies to probe the corresponding biomarkers. The washing solution contained PBS with 0.1% (v/v) Tween-20 (Sigma-Aldrich, USA) (PBST).

### Device fabrication

Prior to the deposition, the glass slides (1 cm × 3 cm) were sonicated in acetone, isopropyl, and deionized (DI) water for 10 min and dried by nitrogen. By simply placing a shadow mask between the target and substrate, a square (5 mm × 5 mm) of dense nanorods was deposited on the glass slides through OAD. The stencils for prepatterning of antibodies on the glass substrates and the microfluidic chips for flow-through assays were both molded in PDMS using conventional soft lithography. A layer of 45-μm-thick SU8 photoresist was patterned on a 4-in. silicon substrate as a template. PDMS elastomer (Sylgard 184, Dow Corning, MI) was poured onto the prepared substrate and cured for half an hour at 120 °C. For cAb conjugated with Au nanorods, the PDMS slab consisted of a 5-mm-long and 100-μm-wide channel with access holes and was placed on top of the nanorod array region. A solution of 0.5 mM DSP was pipetted into the channel of the stencil and incubated for 2 h to form a SAM as an adhesion layer. After extensive rinsing, 10 μg mL^−1^ cAb was introduced into the stencil and incubated in a sealed container for 2 h at room temperature. The stencil was washed with PBST and blocked with 1% BSA before removal. For the dAb attachment, the plain elastomer slab was located on the glass substrate with a bored reservoir 2 mm in diameter overlapping with the predefined dAb deposition zone on glass slides. Then, 10 μg mL^−1^ dAb labeled with fluorescent dye was added and incubated for 2 h at room temperature. Finally, the microfluidic channels were enclosed by permanent bonding over the glass substrates upon activating PDMS surfaces in oxygen plasma (29.6 W Harrick Plasma, 20 s), while the immobilized dAb and the nanorod region on the glass slides were aligned with the dAb deposition zone and reaction chamber patterned in PDMS microfluidic chips under the microscope, respectively. The power during the time of oxygen plasma was delicately adjusted so that the biological activity of predeposited antibodies was unaffected. A conceptual illustration of the fabrication process is schematically described in Supplementary Fig. S5.

### Experiments

After bonding between the microfluidic PDMS cover and glass substrates, each device was placed under an epifluorescence microscope (FN1, Nikon) equipped with a ×20/0.3 NA objective lens (Carl Zeiss) and a filter cube set suitable for the excitation and detection of fluorescent bands (ex/em 493/519 nm). The flow-through immunoassay was performed by pipetting 20 μL of human serum into the sample inlet. The target analyte was directed along the flow path, conjugated with fluorescent dAb, and finally captured by the cAb on Au nanorods. The fluorescent strength of the reaction chamber was imaged using a CCD camera (RT3Mono, SPOT) mounted on the microscope and analyzed through image processing software (ImageJ, NIH, Bethesda). The generated fluorescence intensities were acquired from the region of interest (∼20 μm × 20 μm) and fitted to a five-parameter logistic model to determine the detection limits and dynamic ranges of the assay.

### Simulation

A wave optics module of COMSOL Multiphysics v5.3 (Comsol Inc., Burlington, MA) was used to simulate the electromagnetic field distribution across the device. Electric fields were calculated by constructing cylindrical nanorod arrays with various diameters and spacings on glass substrates (refractive index of 1.5). The remaining domain was set to be water (refractive index of 1.33). A 10-nm-thick perfectly matched layer was utilized at the boundaries of the device. For the light source, a plane wave at *λ* = 493 nm from the device top was normally incident on the substrate. The effect of fluorescence enhancement was calculated based on the Helmholtz wave equation:

$$\nabla \times \left( {\nabla \times E} \right) - k_0^2\varepsilon _{\mathrm{r}}E = 0$$

where *k*_0_ is the wavenumber and *ε*_r_ is the relative permittivity. The permittivity values for metals were adopted from Johnson and Christy’s work^48^.

## Supplementary information

the revised version of supplementary information
