# Supplementary material for: Monolithic integration of nanorod arrays on microfluidic chips for fast and sensitive one-step immunoassays
Source: Microsyst Nanoeng. 2021 Aug 17;7:65. doi: 10.1038/s41378-021-00291-w (PMC8433357; doi:10.1038/s41378-021-00291-w)
Supplement: Supplementary file 1 — the revised version of supplementary information [file 41378_2021_291_MOESM1_ESM.docx]

**SUPPLEMENTARY MATERIAL**

**Monolithic integration of nanorod arrays on microfluidic chips for fast and sensitive one-step immunoassays**

Ye Wang^1^, Jiongdong Zhao^1^, Yu Zhu^3^, Shurong Dong^1,2^, Yang Liu^1^, Yijun Sun^1^, Liling Qian^4^, Wenting Yang^5^ and Zhen Cao*^1,2^

^1^College of Information Science and Electronic Engineering, Zhejiang University, Hangzhou 310027, P.R. China.

^2^Hangzhou Global Scientific and Technological Innovation Center, Zhejiang University, Hangzhou 310018, P.R. China.

^3^Suzhou Institute of Nano-Tech and Nano-Bionics, Chinese Academy of Sciences, Suzhou 215123, P.R. China.

^4^Children’s Hospital of Fudan University, Shanghai, P.R. China.

^5^Genenexus Technology Corporation, Shanghai, P.R. China.

*Correspondence: Zhen Cao ([eezcao@zju.edu.cn](mailto:eezcao@zju.edu.cn))

*
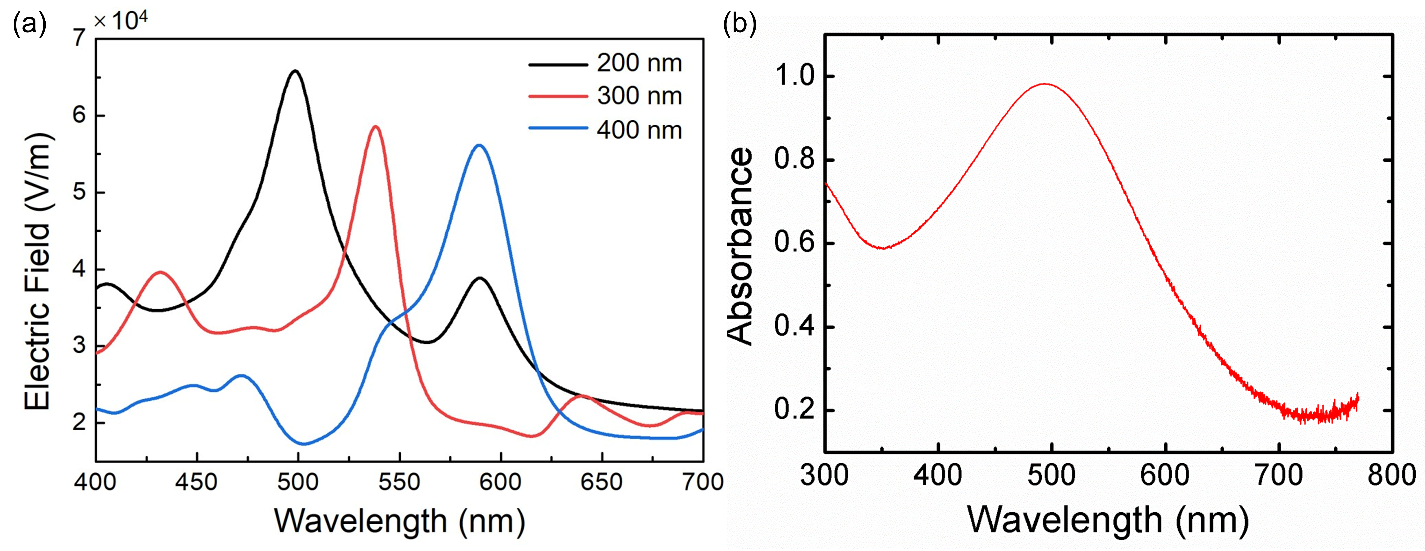
*

***Fig. S1.*** *(a) The simulated* *spectrum of maximum electric fields for Au nanorod arrays in 75-nm diameter but with various pitches. (b) The measured absorbance spectrum of the Au nanorod arrays fabricated through OAD. The nanorods result from an evaporation of the nominal thickness 1.5-μm with an average 75-nm diameter, 600-nm length and 200-nm spacing.*

*
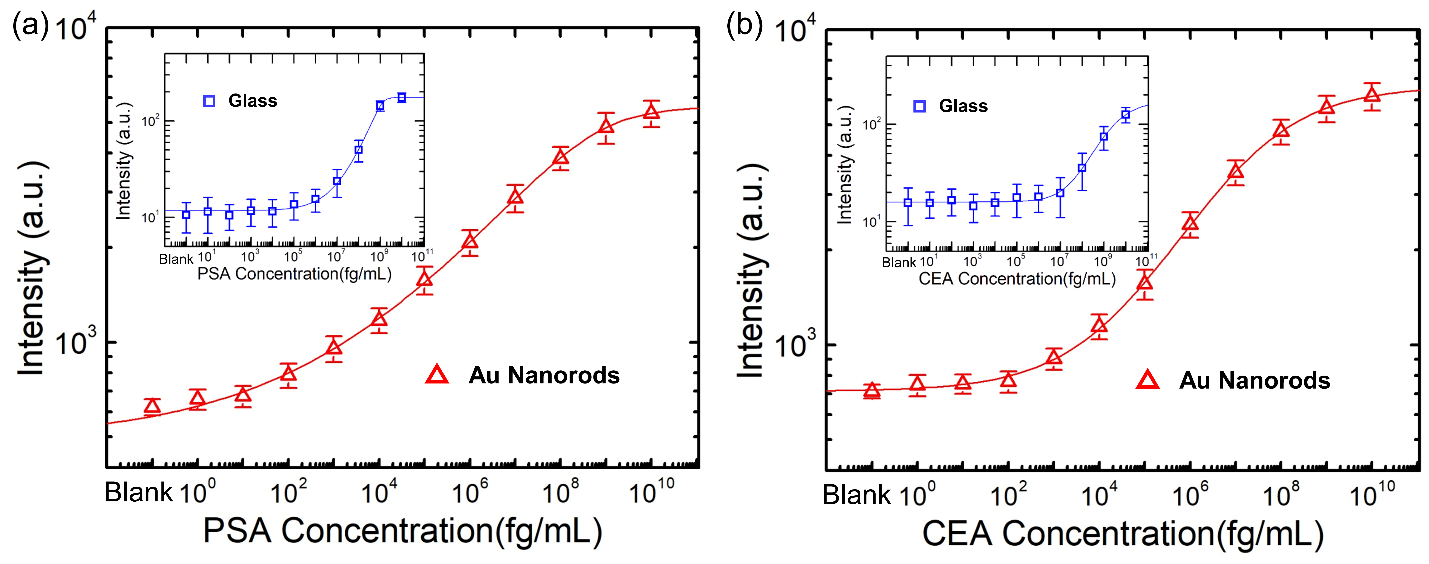
*

***Fig. S2.*** *Fluorescence calibration curve showing the relationship of fluorescent intensity with respect to the concentration of target PSA and CEA on Au nanorods and glass plates. The curves are fitted to 5-parameter logistic non-linear regression models with an excellent agreement (R^2^=0.998). The error bars indicate the standard errors of the respective mean values based on five measurements.*

*
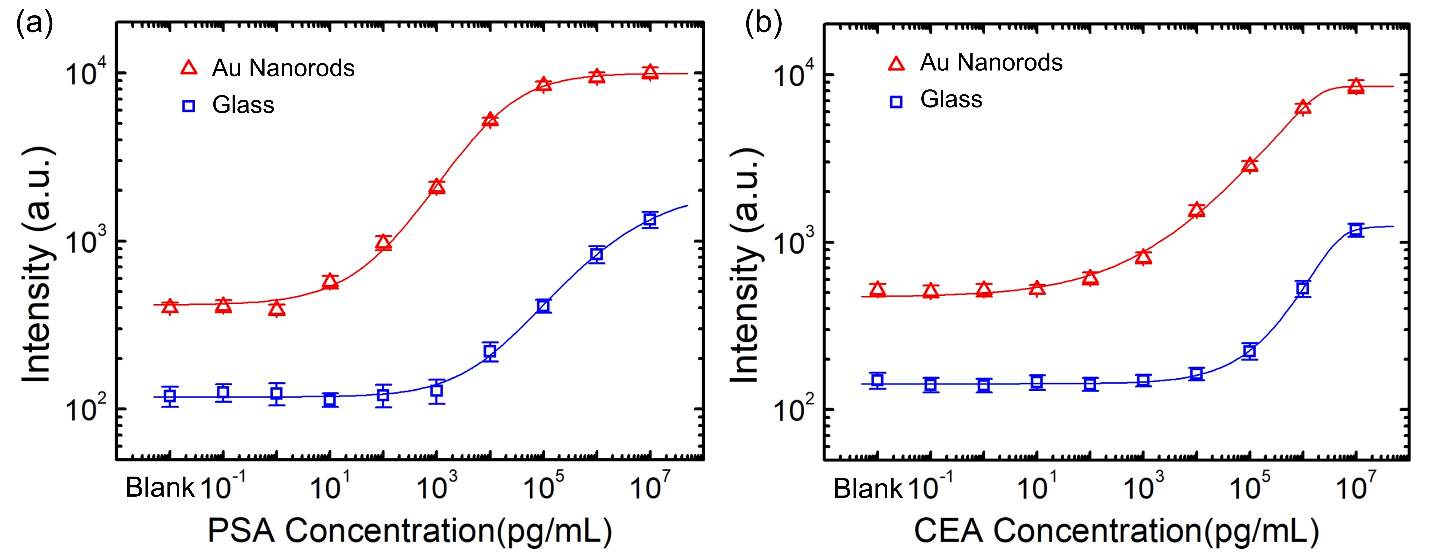
*

***Fig. S3.*** *The calibration curves of PSA and CEA detection using flow-through immunoassay devices with integrated nanorod arrays and glass substrates. The curves are fitted to 5-parameter logistic non-linear regression models. The error bars indicate the standard errors of the respective mean values based on five measurements.*

*
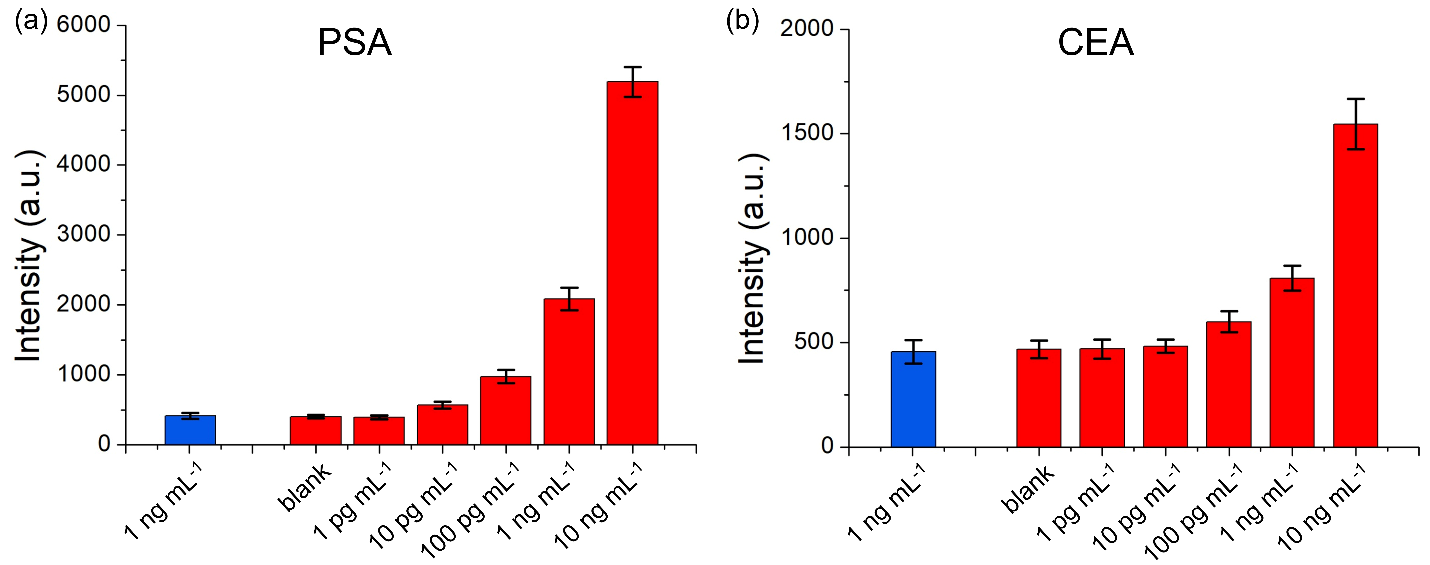
*

***Fig. S4.*** *The graphs show (a) the fluorescent intensities of the flow-through devices for PSA assays by introducing different concentrations of PSA or CEA (1 ng mL^−1^, first column bar) and (b) the fluorescent intensities of the flow-through devices for CEA assays by introducing different concentrations of CEA or PSA (1 ng mL^−1^, first column bar).*

*
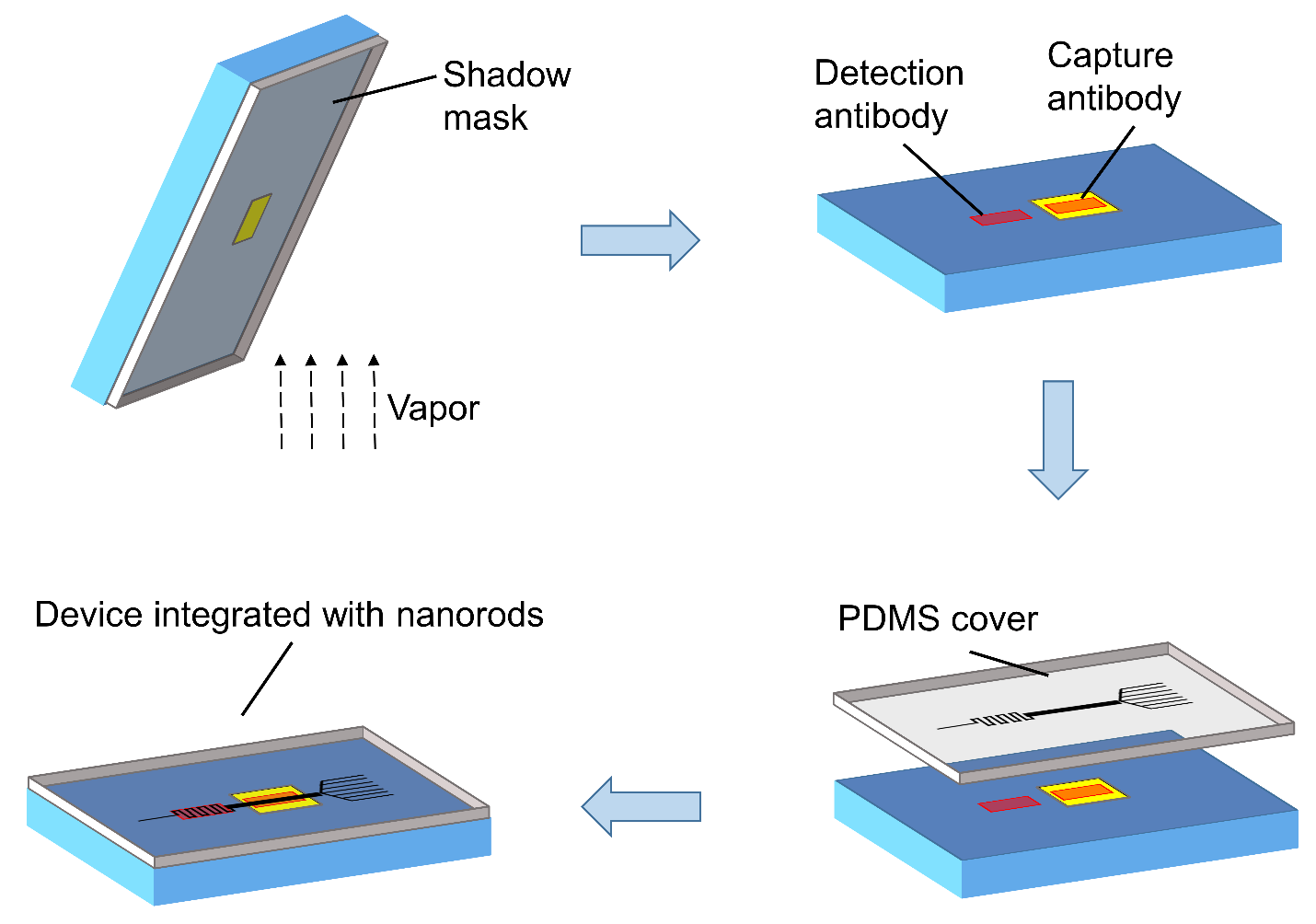
*

***Fig. S5.*** *Illustrations of key fabrication steps of the flow-through immunoassay device integrated with Au nanorods using OAD.*
